# Automated conversion of Millennium‐120 VMAT plans to HDMLC geometry: Software development and treatment of first patients

**DOI:** 10.1002/acm2.13598

**Published:** 2022-03-31

**Authors:** Ray Yang, Michael Lamey, Leigh Bartha, Michael Johnston, Alexandra Warburton, Dawn Gillund, Nathan Becker

**Affiliations:** ^1^ Department of Medical Physics BC Cancer ‐ Centre for the Southern Interior Kelowna British Columbia Canada; ^2^ Department of Radiation Therapy BC Cancer ‐ Centre for the Southern Interior Kelowna British Columbia Canada; ^3^ Department of Computer Science, Mathematics, Physics and Statistics The University of British Columbia Okanagan Kelowna British Columbia Canada

**Keywords:** automation, backup, HDMLC, interoperability, scripting

## Abstract

**Purpose:**

To provide plan backup resiliency for patients treated on a solitary high definition multileaf collimator (HDMLC) linac by developing a fully integrated Eclipse script, which converts patient plans initially optimized on Millennium‐120 (M120) MLC to dosimetrically equivalent leaf motions for delivery on HDMLC. In the event of HDMLC machine downtime, affected patients can be transferred to Millennium‐120 units, and their backup plan delivered without delay.

**Methods:**

Write‐enabled Eclipse scripting is leveraged to generate HDMLC treatment fields with control points parameterized to mimic apertures of an existing Millennium‐120 VMAT plan. Non‐parity between intermediate control point gantry angles of script generated arcs relative to VMAT is reconciled through an interpolation subroutine to correct for the apertures and monitor units that would have existed at intermediate angles. Differences in dosimetric leaf gap are corrected by displacing the subset of leaves undergoing dynamic motion. A nominal change to plan normalization corrects for remaining discrepancies between beam models.

**Results:**

Over 220 non‐SABR VMAT patients were treated on a solitary HDMLC linac with plans converted using the developed script. All have undergone streamlined RO review and physics quality assurance (QA), where the converted plan replicates the original leaf patterns, representing a minor dosimetric perturbation. Analyzing a subset of converted plans delivered at four anatomical sites, on average 99.3% of points pass the 1%/1 mm gamma criterion. Dose‐volume histograms between the original and converted plans are in excellent agreement. ArcCheck measurements comparing delivery of the converted HDMLC plan to the calculated M120 dose distribution averaged a gamma pass rate of 99.4% (95.2%) at a 3%/3 mm (2%/2 mm) criterion. The conversion process takes 30 s to run, avoids errors in exporting/re‐importing, and generates leaf motions deliverable within machine limits.

**Conclusion:**

The methodology developed for automated plan conversion helped maximize the utilization of a solitary HDMLC linac, while preserving backup interoperability with minimal overhead.

## 1 INTRODUCTION

Any radiotherapy center operating with mixed linac MLC configurations can appreciate the challenges that arise during inevitable machine downtime, as well as the benefits of linac plan interoperability. Furthermore, as deployment of linac‐based stereotactic irradiation spreads to smaller centers, these facilities may be required to operate a solitary high definition multileaf collimator (HDMLC) linac, where the impact of downtime can be particularly acute.

Recent experience at one center in our province involved significant downtime of their solitary HDMLC linac during the first months of operation. This downtime was disruptive to patients, required time consuming replans, and resulted in the machine subsequently being underutilized to mitigate risk. Another provincial center continues to operate a solitary HDMLC linac and dedicates significant resources double‐planning cases to ensure that patients can be transferred in case of downtime. With growing patient populations and wait times, efficient utilization of all treatment resources is also an important consideration.

Our regional cancer center serves a large geographic catchment spanning over 110 000 km^2^. Patients in this catchment often travel long distances (>100 km) for treatment and pay out of pocket for local accommodations. With the introduction of a solitary HDMLC linac alongside four existing linacs equipped Millennium‐120 MLC, leadership sought to maximize institutional preparedness for patients to be treated on time, specifically by minimizing disruptions in the event of downtime while maintaining full utilization of treatment resources. This required either (1) creating plans exclusively for the HDMLC in advance of an uncertain clinical release date without a backup, or (2) resource intensive double‐planning for HDMLC and M120 thereby preserving a backup, though also doubling the work to generate, review, and QA two independent plans.

One vendor solution, which comes at an additional cost, is Varian's dose‐volume histogram (DVH)‐based plan converter tool.^1^ The process involves stochastically re‐optimizing the plan on a different MLC to replicate the original plan's DVH. However, past studies have shown inconsistent performance and reduced conformity.^2^, ^3^ Moreover, re‐optimization produces different leaf patterns, which in our opinion would require greater effort for review and quality assurance (QA) as it represents a completely new treatment plan.

In exploring options for plan backup resiliency during the transition to a center utilizing one HDMLC in a mixed MLC environment, we hypothesize benefit in developing a method to maintain the physical attributes of an original M120 plan when converting to a dosimetrically equivalent HDMLC delivery while also correcting for small dosimetric differences between the MLC types.

By leveraging write‐enabled scripting capabilities in Eclipse, we automate remapping of leaf positions from an existing M120 plan onto HDMLC geometry, recreating the same beam apertures, such that both plans can be reviewed in streamlined fashion. Compared to other scripting attempts in the literature,^4^, ^5^ our solution is fully integrated with the Eclipse planning system, which benefits ease of clinical adoption, does not require export/re‐import of plan files, and ensures the resulting plans are deliverable within machine limits.

## 2 METHODS

### 2.1 Comparing MLC geometries

**FIGURE 1:**
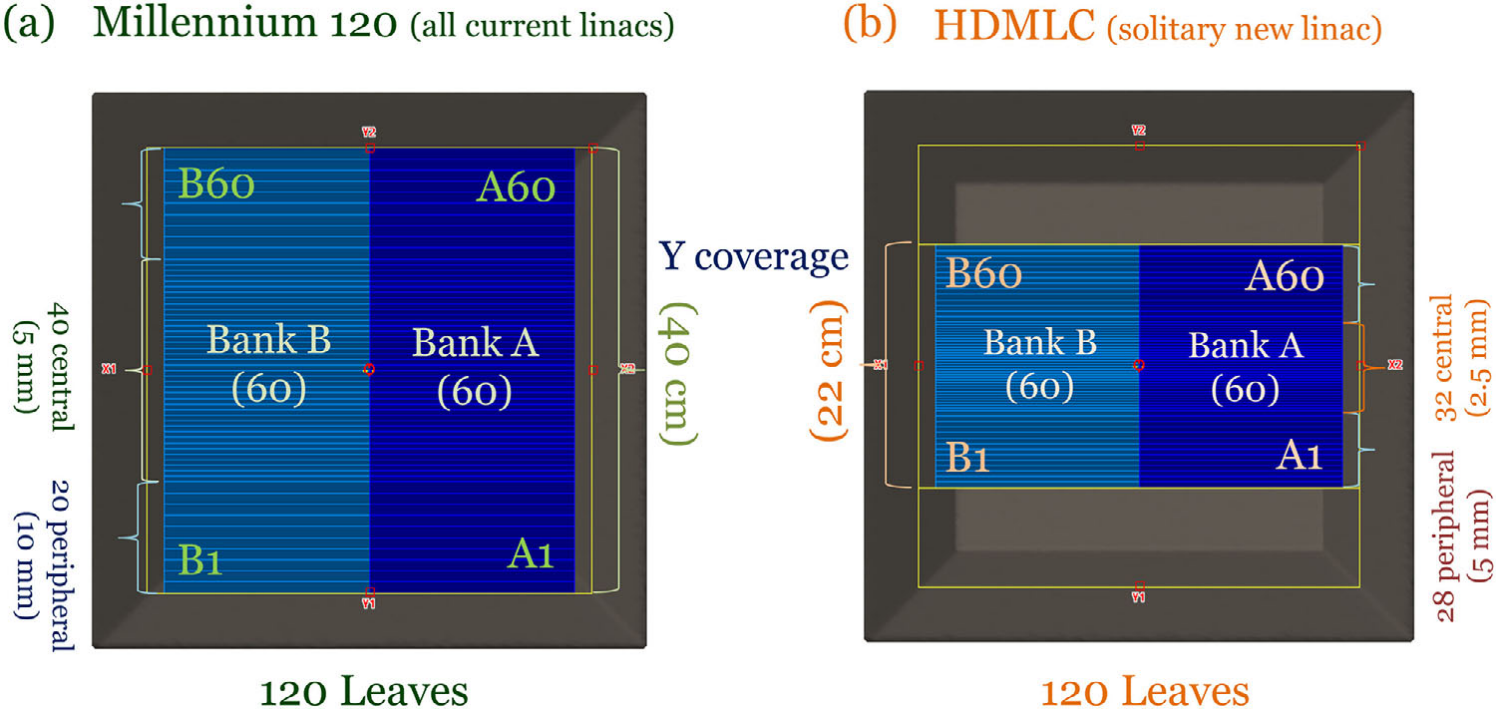
(a) Millennium‐120 and (b) high definition multileaf collimator (HDMLC) geometries

**FIGURE 2:**
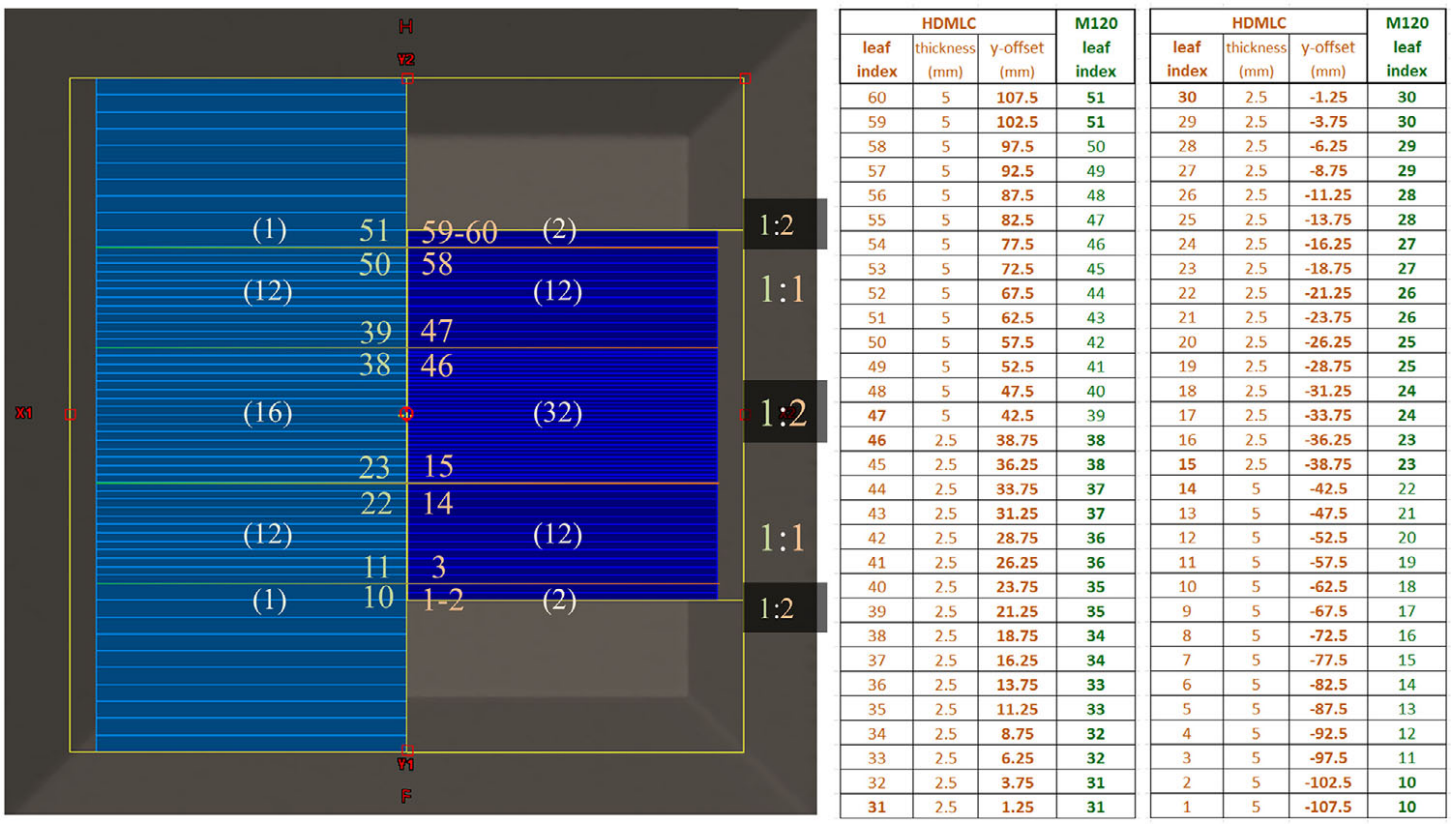
Deterministic mapping convention from Millennium‐120 to high definition multileaf collimator (HDMLC)

Both Millennium‐120 and HDMLC have 120 leaves (60 per bank) as illustrated in Figure 1. The central pairs offer finer widths (5 mm for M120, 2.5 mm for HDMLC), while peripheral leaves are coarser (10 mm for M120, 5 mm for HDMLC). In each grouping, HDMLC leaves are narrower by a factor of 2. This has implications on the maximum field size accommodated in the y‐direction (40 cm for M120 vs. 22 cm for HDMLC). For a plan to be deliverable on HDMLC, neither Y1 nor Y2 jaws should exceed 11 cm; however, 10.5 cm is recommended to provide enough margin of MLC shielding to attenuate off‐axis out‐of‐field dose scattering off the primary collimator.^6^ The majority of clinical plans meet this constraint. Any in‐field leaf pattern shaped by the M120 can be replicated using the HDMLC according to conventions in Figure 2. For example, in the region −4cm≤y≤4cm, each 5‐mm M120 leaf can be replicated by pairing adjacent 2.5‐mm HDMLC leaves extended the same amount.

Although seemingly trivial to remap leaf positions, such functionality is not supported in the Eclipse planning system. Changing from M120 to HDMLC on an optimized plan will delete all leaf positions, requiring a replan. As a workaround, the scripting interface in Eclipse offers a way to capture the control points (snapshots or keyframes through the delivery progression) and model these on a new treatment field. Each control point encodes the gantry angle, meterset weight (percentage of total monitor units), jaw positions, and MLC leaf positions. Given a beam model, prescription dose, and control points, these parameters uniquely define the treatment plan. This applies to any delivery technique, including volumetric modulated arc therapy (VMAT), IMRT, or Field‐in‐Field, but the focus herein will concern VMAT plans, since they represent the most complex use case, and highest workload for our new HDMLC linac.

### 2.2 Eclipse scripting capabilities and limitations

The Eclipse treatment planning system includes a C# scripting interface (ESAPI). Version 15 and above allow users to write to the database. In particular, a new arc is generated using the method^7^:

AddVMATBeam(machineParameters, metersetWeights, collimatorAngle, gantryStart, gantryStop, gantryDirection, isocenterCoordinates)

In theory, plan conversion is trivial as changing each subfield from M120 to HDMLC then re‐indexing the leaf extensions according to a deterministic mapping (Figure 2); however a central non‐triviality arises due to nonparity between control point angles.

For a script‐generated arc, parameterized by a given gantry start and stop angle, the location of individual control points is a read‐only parameter, usually instantiated as uniform 2° increments. In contrast, a plan that has gone through VMAT optimization does not contain evenly spaced control points, rather undergoes distinct phases of acceleration (increasing angular spacing) at the start of the arc and deceleration (decreasing angular spacing) near the end of the arc.

**FIGURE 3:**
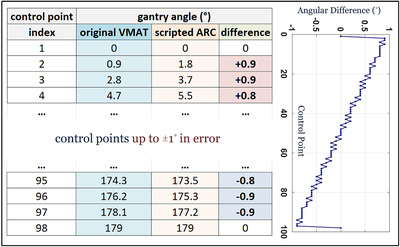
Differences in control point gantry angles between VMAT optimized and script generated arc plans with the same start and stop angle

**FIGURE 4:**
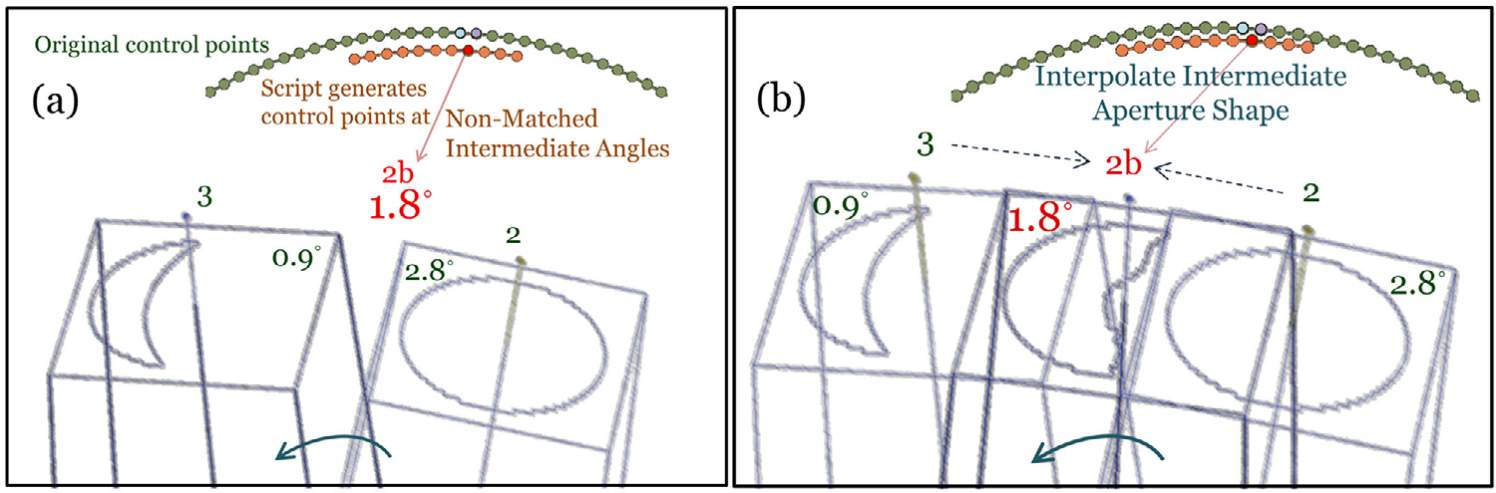
(a) Schematic illustrating uncertainty of placing nonmatched control point angle between changing apertures; (b) Interpolating leaf motions to generate high definition multileaf collimator (HDMLC) aperture that would have existed at intermediate control point angle

Given the same start and stop angles, the difference between intermediate control point angles of a script generated arc compared to VMAT can be up to ±0.9° as shown in Figure 3 and conceptually illustrated in Figure 4. Moreover, the scripting environment enforces the gantry angle at each control point as a read‐only parameter, which cannot be modified to match the original VMAT plan. Despite preserving the same start and stop angles, as well as total number of control points, simply propagating remapped leaf positions from the original M120 VMAT plan to the perturbed control point angles of a scripted arc would be erroneous because it could be aimed at an angle up to ±0.9° in deviation. Likewise, propagating the same meterset weights to perturbed control point angles would introduce nontrivial systematic errors in dose rate modulation.

### 2.3 Interpolation subroutine

Given the goal to replicate the original M120 leaf sequence to generate a dosimetrically equivalent arc delivery on HDMLC, our solution involved interpolation to determine the aperture shape, meterset, and jaw positions that would exist at the intermediate non‐matched control point angles. Figure 4 conceptually illustrates an interpolated aperture shape at script‐generated point 2b (at 1.8) based on neighboring source control points at 2.8° and 0.9°.

For each arc‐based treatment field, AddVMATBeam is initially run to generate a “scout field” yielding the precise number and spacing of control points expected in a script generated arc given the same start and stop angles. This information is used to generate interpolation factors, which specify the relative contributions of nearest neighbor control points from the original VMAT arc. We then calculate corrected meterset weights, jaw positions, and leaf positions (both its index according to Figure 2 and appropriate extension for the intermediate control point angle) as they would exist at the intermediate gantry angle to mimic the original M120 plan.

AddVMATBeam is run a second time to generate the HDMLC treatment field, initialized with corrected parameters corresponding to the exact control point angles it is known to generate. With these corrections, the resulting HDMLC plan is as close as possible to reconstructing the original M120 delivery. Eliminating systematic errors pave way for reliable corrections of other dosimetric factors, which may vary between machines.

### 2.4 Dosimetric leaf gap correction

**FIGURE 5:**
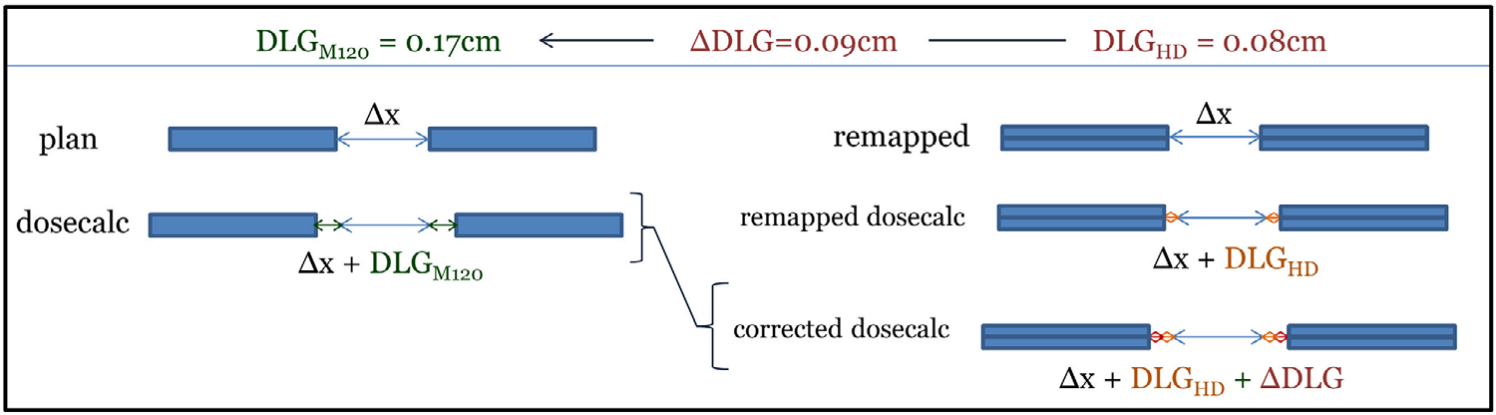
Dosimetric leaf gap (DLG) correction between source and deployment units

The rounded ends of MLC leaves are not explicitly modeled by the treatment planning system. In highly modulated deliveries, differences between the calculated and delivered doses can be reconciled by a dosimetric leaf gap (DLG) correction unique to each MLC design and beam energy. Commissioning measurements revealed a narrower DLG for HDMLC compared to M120. Therefore an additional geometric correction to replicate the original plan's dosimetric characteristics involved perturbing the remapped leaf positions by the difference in DLG, as illustrated in Figure 5.

There may arise solutions in VMAT optimization where narrow apertures on the order of 0.5 mm are present within the field over several consecutive control points, mainly with the intent of shielding. We do not expand the DLG in these instances to avoid unnecessary extra dose.

Often the original M120 VMAT plan is already operating near machine limits, therefore perturbing leaf positions for DLG correction has the potential to generate errors associated with carriage group limits, leaf overtravel, and minimum dynamic leaf gap. Specifically, only leaves undergoing motion, with a gap exceeding 0.6 mm are extended by the DLG correction, only if doing so does not exceed carriage group limits. In this way, deliverability is maintained.

Remaining differences in dosimetric output of machines could arise due to MLC leaf transmission, inter‐leaf leakage, linac photon spectrum, head scatter output factors, mean radial energy as a function of off‐axis displacement, collimator backscatter factor, and source dimensions. A small change to plan normalization corrects for remaining discrepancies between beam models.

For clarity, the full source code implementation is provided in Supporting Information as Appendix A.

## 3 RESULTS AND DISCUSSION

### 3.1 Comparing dose distributions between original M120 and converted HDMLC plans

So far, more than 220 non‐SABR (stereotactic ablative body radiosurgery) VMAT patients have been treated on a solitary HDMLC linac with a converted plan generated by the script. The original M120 plan serves as a backup, however both variants are approved by a radiation oncologist (RO) and physicist, indicating their assessment that the plans are dosimetrically equivalent.

**FIGURE 6:**
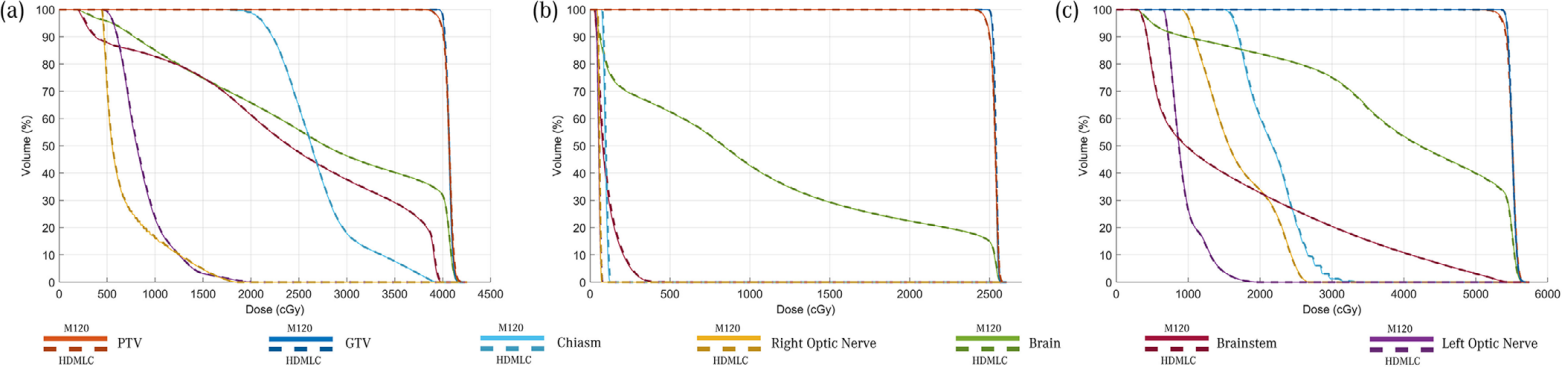
Dose‐volume histograms (DVH) comparison between original M120 plan (solid) versus script converted high definition multileaf collimator (HDMLC) plan (dotted) for central nervous system (CNS) site across three representative patients (a, b, and c)

**FIGURE 7:**
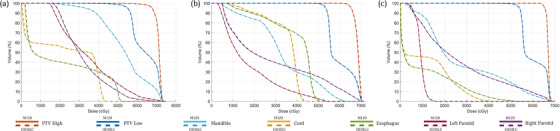
Dose‐volume histograms (DVH) comparison between original M120 plan (solid) versus script converted high definition multileaf collimator (HDMLC) plan (dotted) for head and neck (HN) site across three representative patients (a, b, and c)

**FIGURE 8:**
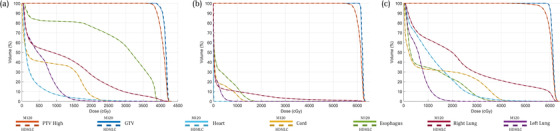
Dose‐volume histograms (DVH) comparison between original M120 plan (solid) versus script converted high definition multileaf collimator (HDMLC) plan (dotted) for lung site across three representative patients (a, b, and c)

**FIGURE 9:**
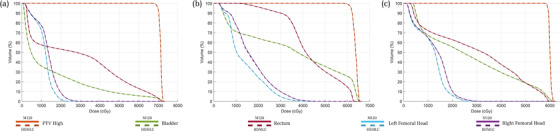
Dose‐volume histograms (DVH) comparison between original M120 plan (solid) versus script converted high definition multileaf collimator (HDMLC) plan (dotted) for prostate site across three representative patients (a, b, and c)

As a quantitative audit of the process, we examine three random patients at each anatomical site, consisting of central nervous system, head and neck (HN), Lung, and Prostate. Their DVHs are presented in Figures 6, 7, 8, 9, respectively. Across all sites, the script converted HDMLC DVH (plotted as dotted lines) appears to overlap with the original M120 DVH (plotted as solid lines). This applies to the target volume and key organs at risk. Occasionally at very low doses (on the order of 10% of prescription), the HDMLC DVH deviates slightly higher (most evident in Figure 7a for the spinal cord in a HN case). Likely this is due to cumulative effects of inter‐leaf leakage from an increased number of narrower HDMLC leaves subtending the same longitudinal extent.

Investigators studying Varian's DVH‐based plan converter have found far greater variability between source and converted plan DVHs, especially for organs at risk. These manifest as distinct nonoverlapping slopes, which cannot be reconciled with a change to plan normalization.^3^ Such variability can be expected for a process based on stochastic re‐optimization, whereas our process based on deterministic remapping of beam apertures can be treated as a dosimetric perturbation preserving the DVH of the original plan, with examples across Figures 6, 7, 8, 9.

For the same patient cohort, column 2 of Table 1 presents gamma analysis between the original and converted plans. For most plans, >99% of voxels pass the 1%/1 mm global Van Dyk gamma criterion. The worst agreement around 97% was observed for a HN case comprised of three highly modulated arcs, with Y‐field sizes close to the limits of the HDMLC. It is important to note, achieving an extremely high gamma pass rate is not a prerequisite for both plans to be approved as clinically viable. One factor affecting gamma is the plan normalization, shown in columns 3 and 4 of Table 1. Despite interpolating to the correct control point apertures, metersets, and adjusting for differences in DLG, residual differences remain in the beam models. This is easily rectified by changing the global plan normalization by approximately 0.5%, without explicit attempts to optimize its value.

**TABLE 1:** Comparing dose distributions between original M120 and converted high definition multileaf collimator (HDMLC) plans

		Dose comparison	Plan normalization		Conformity index		Heterogeneity index	
Patient	Site	γ 1%/1 mm th10%	M120	HD	M120	HDMLC	M120	HDMLC
**1**	**CNS**	98.75	100	99.7	92.57	92.09	3.63	3.78
**2**	**CNS**	99.82	100	99.5	90.44	90.02	2.96	3.16
**3**	**CNS**	99.53	100	99.6	94.04	93.12	3.84	4.02
**4**	**HN**	97.22	100	99.4	82.74	82.03	7.16	7.48
**5**	**HN**	99.09	100	99.4	91.64	92.33	7.04	7.17
**6**	**HN**	99.58	100	99.3	90.51	90.34	3.58	3.75
**7**	**Lung**	99.99	100	99.8	73.38	73.95	8.78	8.94
**8**	**Lung**	99.99	100	100	81.92	81.72	6.46	6.82
**9**	**Lung**	99.95	100	99.7	70.87	70.75	8.77	8.84
**10**	**Pros**	99.99	100	99.8	93.29	92.60	3.25	3.38
**11**	**Pros**	98.72	99.6	99.2	95.34	95.32	5.52	5.52
**12**	**Pros**	99.99	100.1	99.9	52.44	51.84	2.97	3.10

Abbreviations: CNS, central nervous system; HN, head and neck.

During plan review, the two variants can be considered independently, both as being viable and meeting clinical constraints, yet rendering the same underlying leaf motions such that any residual differences no longer the dominant source of uncertainty in choosing which variant to deliver on the patient.

In Table 1, we also compare quality metrics of the conformity index and heterogeneity index, commonly defined as^8^ :

$$\mathrm{CI}\;=\;100\cdot \frac{V_{100\%}\;\text{within}\;\text{PTV}}{V_{\mathrm{PTV}}}$$

$$\mathrm{HI}\;=\frac{\left(D_{5\%}-D_{95\%}\right)}{D_{\mathrm{pres}}}\;$$

The process of plan conversion slightly reduces coverage of the 100% isodose, while slightly increasing dose heterogeneity within the PTV. The discrepancy is of sufficiently low magnitude to be clinically insignificant.

ArcCheck phantom measurement is routinely used for patient‐specific quality assurance of VMAT plans. The first batch of HDMLC patients was transferred from existing treatments on M120 units, providing a unique opportunity for direct comparison of measurements on both machines, as shown in columns 3 and 4 of Table 2.

**TABLE 2:** ArcCheck measurement of converted high definition multileaf collimator (HDMLC) plan compared to M120 measurement and M120 planned dose

		ArcCheck measurement of converted HDMLC plan compared to			
		ArcCheck measurement of original M120 plan		M120 planned dose	
		% of points within dose		γth 10%	
**Patient**	**Site**	2%:	1%:	3%/3 mm:	2%/2 mm:
1	Pelvis	98.8	93.6	99.9	98.5
2	H&N	99.0	95.8	99.9	96.9
3	H&N	89.6	72.6	99.9	95.4
4	H&N	97.5	89.9	99.4	98.3
5	Pelvis	97.3	87.4	100	98.4
6	H&N	99.6	93.6	98.2	92.2
7	Pelvis	100	98.2	98.3	84.5
8	H&N	98.9	95.8	99.9	97.3
	**Average**	**97.59**	**90.86**	**99.44**	**95.19**

Abbreviation: H&N, head and neck.

Despite the potential for geometric errors in phantom alignment and inherent dosimetric differences between two unique machines, the plan conversion script facilitated measurements where on average 97.6% (90.9%) of diodes detected dose values within 2% (1%).

**FIGURE 10:**
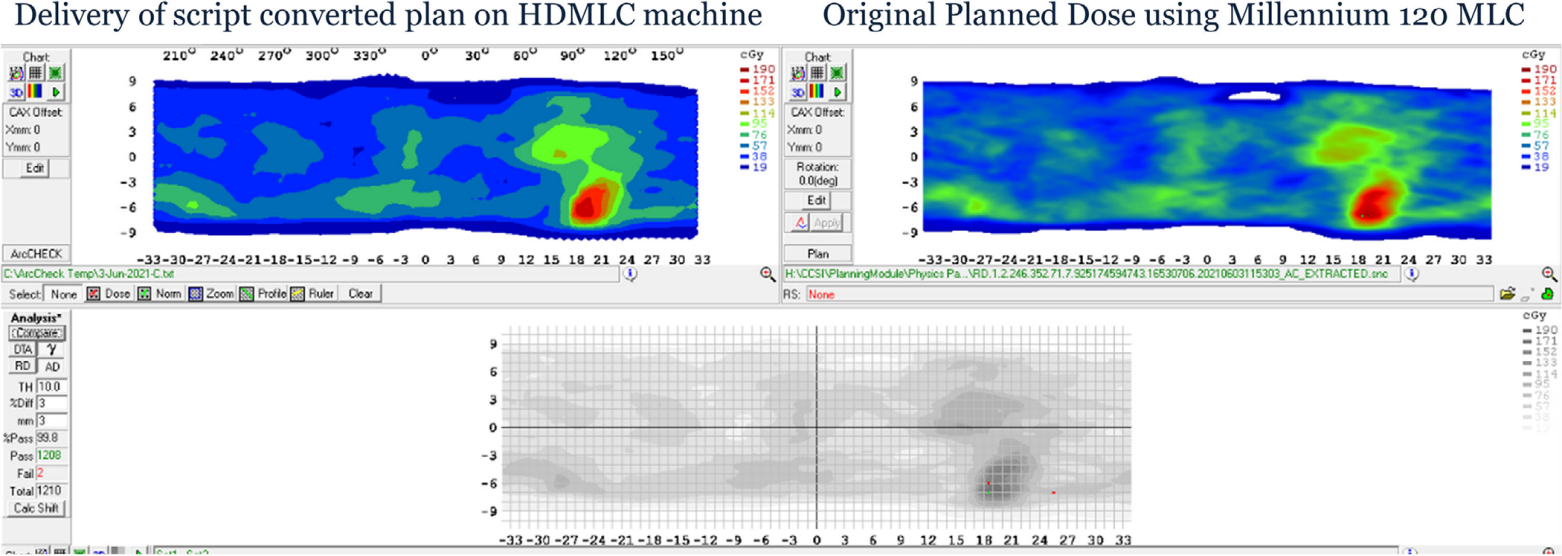
Example of converted high definition multileaf collimator (HDMLC) plan delivered on ArcCheck compared to dose calculated on M120

A more forgiving metric, shown in columns 5 and 6 of Table 2, considers gamma comparison between measured dose delivered by the converted plan on an HDMLC linac to the M120 planned dose. On average 99.4% (95.2%) of points pass the 3%/3 mm (2%/2 mm) criterion. An example is shown in Figure 10 for HN site in the Sun Nuclear Corporation (SNC)‐patient software.^9^

These measurements establish confidence that a deterministic paradigm for plan conversion (leaf index remapping, interpolation of leaf extension/meterset weights at intermediate control points, followed by selective DLG correction and nominal change to plan normalization) is able to preserve the original plan's dosimetric characteristics.

**FIGURE 11:**
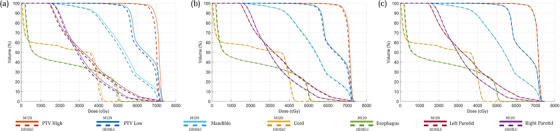
Dose‐volume histograms (DVH) comparison between script converted high definition multileaf collimator (HDMLC) plan versus original M120 VMAT plan for the same head and neck patient (a) without correcting for dosimetric leaf gap (DLG) or plan normalization, (b) with DLG correction, but no plan normalization, (c) with DLG adjusting plan normalization by 0.6%

To illustrate the impact of DLG correction and plan normalization, Figure 11a presents a DVH comparison where both corrections are omitted. Dotted and solid plots are clearly separated, with the HDMLC variant appearing underdosed. Figure 11b reintroduces the DLG correction, which brings DVH plots much closer and improves conformity of the HDMLC variant. A final perturbation to plan normalization by 0.6% brings the DVH plots to overlap in Figure 11c.

### 3.2 Changes to clinical process

**FIGURE 12:**
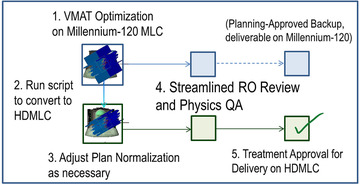
Overview of planning workflow

Since plan conversion is only intended as one‐way from M120 plans to HDMLC plans, inverse optimization is initially performed on a Millennium‐120 linac. This excludes plans that might clinically benefit from the finer resolution of the HDMLC leaves, most notably SRS and SABR plans. Standard VMAT plans comprise a significant proportion of treatment workload (>2/3) on the HDMLC machine. Since each patient returns to be treated 20–35 fractions, our department leadership prioritized having a backup plan in place based on an original M120 plan. For the vast majority of sites, M120 plans are clinically acceptable and used as a starting point for this procedure. Once a satisfactory M120 plan is produced, the script is run to generate an equivalent HDMLC plan, which takes about 30 s. Finally, the dose is calculated on the new HDMLC plan. After reviewing DVH objectives, plan normalization is adjusted as necessary (typically by 0.5%) to match the two plans. The RO then reviews both plans, setting them to a reviewed state to minimize turnaround time should the backup plan be needed. Both plans are sent to Physics for independent QA and plan checks. An overview of the process is shown in Figure 12.

In terms of additional workload, running the script and preparing a backup plan add approximately 5 min in total, compared to several hours it would take to produce a uniquely independent plan.

Review is expedited since both are mimicking the same leaf patterns such that by examining one set of leaf motions in detail, there can be a high degree of confidence that the converted plan is delivering the same beamlets, requiring less time to QA than a true replan where the leaf motions and dose to organs can be completely different. In the event of machine hardware failure, fallback to a dosimetrically nearly identical plan can be delivered on the remaining units without delaying the patient or waiting for a replan.

## 4 CONCLUSIONS

An efficient solution to provide plan backup interoperability was developed in the form of a write‐enabled Eclipse script, which automates conversion of VMAT plans initially optimized on Millennium‐120 MLC to dosimetrically equivalent leaf sequences for delivery on HDMLC. By correcting for the DLG and interpolating control point parameters to their proper phase of progression, excellent dosimetric equivalence was achieved, enabling streamlined review of both plans as dosimetric perturbations of one another. Full integration with the Eclipse scripting interface streamlined clinical adoption with minimal overhead and ensured deliverability of the converted plans.

## CONFLICT OF INTEREST

The authors declare that there is no conflict of interest that could be perceived as prejudicing the impartiality of the research reported.

## AUTHOR CONTRIBUTIONS

Ray Yang: Algorithm and software development, dosimetric validation, drafted manuscript and figures. Michael Lamey: Insights on dosimetric leaf gap, eclipse scripting, and clinical plan checks, and reviewed manuscript. Leigh Bartha: Process development for planning, tested on prospective patient plans, and reviewed manuscript. Michael Johnston: Insights from planning, planned and converted first HDMLC patients for clinical treatment, identified challenging edge cases to refine algorithm, and reviewed manuscript. Alexandra Warburton: Prepared and treated first patients on HDMLC linac, compiled statistics on machine workload, insights on downtime protocols, and reviewed manuscript. Dawn Gillund: Prepared and treated first patients on HDMLC linac, insights on patient experience and setup, and reviewed manuscript. Nathan Becker: Project conception, process development for planning, and reviewed manuscript.

## Supporting information

Supporting Information
