# Supplementary material for: Automated conversion of Millennium‐120 VMAT plans to HDMLC geometry: Software development and treatment of first patients
Source: J Appl Clin Med Phys. 2022 Mar 31;23(6):e13598. doi: 10.1002/acm2.13598 (PMC9195034; doi:10.1002/acm2.13598)
Supplement: Supplementary file 1 — Supporting Information [file ACM2-23-e13598-s001.docx]

**using** System**;**

Appendix A: Full Source Code Implementation

**using** System**.**Linq**;**

**using** System**.**Text**;**

**using** System**.**Windows**;**

**using** System**.**Collections**.**Generic**;**

**using** System**.**Reflection**;**

**using** System**.**Runtime**.**CompilerServices**;**

**using** System**.**Xml**;**

**using** VMS**.**TPS**.**Common**.**Model**.**API**;**

**using** VMS**.**TPS**.**Common**.**Model**.**Types**;**

// Code written by Ray Yang, BC Cancer Centre for the Southern Interior

// June 2021

**[**assembly**:** AssemblyVersion**(**"1.0.0.0"**)]**

**[**assembly**:** AssemblyFileVersion**(**"1.0.0.0"**)]**

**[**assembly**:** AssemblyInformationalVersion**(**"1.0"**)]**

**[**assembly**:** ESAPIScript**(**IsWriteable **=** **true)]**

**namespace** VMS**.**TPS

**{**

**public** class Script

**{**

**public** static int**[]** mapL **=** **{** 9**,** 9**,** 10**,** 11**,** 12**,** 13**,** 14**,** 15**,** 16**,** 17**,** 18**,** 19**,** 20**,** 21**,** 22**,** 22**,** 23**,** 23**,** 24**,** 24**,** 25**,** 25**,** 26**,** 26**,** 27**,** 27**,** 28**,** 28**,** 29**,** 29**,** 30**,** 30**,** 31**,** 31**,** 32**,** 32**,** 33**,** 33**,** 34**,** 34**,** 35**,** 35**,** 36**,** 36**,** 37**,** 37**,** 38**,** 39**,** 40**,** 41**,** 42**,** 43**,** 44**,** 45**,** 46**,** 47**,** 48**,** 49**,** 50**,** 50 **};**

**public** static float eps **=** 150.0f**;**

**public** Script**()**

**{**

**}**

**[**MethodImpl**(**MethodImplOptions**.**NoInlining**)]**

**public** void Execute**(**ScriptContext context**,** Window mainWindow**)**

**{**

string deployUnit **=** **null;**

string nodetext **=** **null;**

float dlg_src_6X **=** 0.17f**;** // default value; cm

float dlg_deploy_6X **=** 0.08f**;** // cm

float dlg_delta_6X **=** 0.0f**;**

float dlg_src_6F **=** 0.14f**;** // cm

float dlg_deploy_6F **=** 0.11f**;** // cm

float dlg_delta_6F **=** 0.0f**;**

float dlg_delta **=** 0.0f**;**

XmlDocument doc **=** **new** XmlDocument**();** // read settings from hills_params.xml

string path **=** System**.**IO**.**Path**.**GetDirectoryName**(**Assembly**.**GetExecutingAssembly**().**Location**)** **+** @"\hills_params.xml"**;**

doc**.**Load**(**@path**);**

XmlNodeList dlgSrcNodeList **=** doc**.**DocumentElement**.**GetElementsByTagName**(**"dlgSrc_6X"**);**

**foreach** **(**XmlNode n **in** dlgSrcNodeList**)**

**{**

**if** **(**n**.**SelectSingleNode**(**"cm"**)** **!=** **null)**

**{**

nodetext **=** n**.**InnerText**;**

**try** **{** dlg_src_6X **=** float**.**Parse**(**nodetext**);** **}**

**catch** **{** **}**

**}**

**}**

XmlNodeList dlgSrcNodeListF **=** doc**.**DocumentElement**.**GetElementsByTagName**(**"dlgSrc_6F"**);**

**foreach** **(**XmlNode n **in** dlgSrcNodeListF**)**

**{**

**if** **(**n**.**SelectSingleNode**(**"cm"**)** **!=** **null)**

**{**

nodetext **=** n**.**InnerText**;**

**try** **{** dlg_src_6F **=** float**.**Parse**(**nodetext**);** **}**

**catch** **{** **}**

**}**

**}**

XmlNodeList deployNodeList **=** doc**.**DocumentElement**.**GetElementsByTagName**(**"deployUnit"**);**

**foreach** **(**XmlNode n **in** deployNodeList**)**

**{**

**if** **(**n**.**SelectSingleNode**(**"name"**)** **!=** **null)**

**{**deployUnit **=** n**.**InnerText**;}**

**}**

XmlNodeList dlgDeployNodeList **=** doc**.**DocumentElement**.**GetElementsByTagName**(**"dlgDeploy_6X"**);**

**foreach** **(**XmlNode n **in** dlgDeployNodeList**)**

**{**

**if** **(**n**.**SelectSingleNode**(**"cm"**)** **!=** **null)**

**{**

nodetext **=** n**.**InnerText**;**

**try** **{** dlg_deploy_6X **=** float**.**Parse**(**nodetext**);** **}**

**catch** **{** **}**

**}**

**}**

XmlNodeList dlgDeployNodeListF **=** doc**.**DocumentElement**.**GetElementsByTagName**(**"dlgDeploy_6F"**);**

**foreach** **(**XmlNode n **in** dlgDeployNodeListF**)**

**{**

**if** **(**n**.**SelectSingleNode**(**"cm"**)** **!=** **null)**

**{**

nodetext **=** n**.**InnerText**;**

**try** **{** dlg_deploy_6F **=** float**.**Parse**(**nodetext**);** **}**

**catch** **{** **}**

**}**

**}**

dlg_delta_6X **=** 10.0f ***** 0.5f ***** **(**dlg_src_6X **-** dlg_deploy_6X**);** // cm -> mm

dlg_delta_6F **=** 10.0f ***** 0.5f ***** **(**dlg_src_6F **-** dlg_deploy_6F**);** // cm -> mm

Patient p **=** context**.**Patient**;**

ExternalPlanSetup planSrc **=** context**.**ExternalPlanSetup**;**

**if** **(**planSrc **==** **null)**

**throw** **new** ApplicationException**(**"Please load source plan."**);**

p**.**BeginModifications**();**

string courseId **=** context**.**Course**.**Id**;**

Course course **=** p**.**Courses**.**Where**(**o **=>** o**.**Id **==** courseId**).**SingleOrDefault**();**

ExternalPlanSetup planNew **=** **(**ExternalPlanSetup**)**course**.**CopyPlanSetup**(**planSrc**);**

**if** **(**planNew**.**Id**.**Length **<** 12**)**

**{**

planNew**.**Id **=** planSrc**.**Id **+** " H"**;**

**}**

**foreach** **(**Beam beamSrc **in** planSrc**.**Beams**)**

**{**

**if** **(!**beamSrc**.**IsSetupField **&&** beamSrc**.**Technique**.**Id**.**Contains**(**"ARC"**))**

**{**

var collimatorAngle **=** beamSrc**.**ControlPoints**.**First**().**CollimatorAngle**;**

var gantryDirection **=** beamSrc**.**GantryDirection**;**

var ganSrc **=** beamSrc**.**ControlPoints**.**Select**(**cp **=>** cp**.**GantryAngle**).**ToArray**();**

var mwSrc **=** beamSrc**.**ControlPoints**.**Select**(**cp **=>** cp**.**MetersetWeight**).**ToArray**();**

var isocenter **=** beamSrc**.**IsocenterPosition**;**

var beamWeight **=** beamSrc**.**WeightFactor**;**

ExternalBeamMachineParameters MachineParameters **=**

**new** ExternalBeamMachineParameters**(**deployUnit**,** "6X"**,** beamSrc**.**DoseRate**,** beamSrc**.**Technique**.**Id**,** string**.**Empty**);**

**if** **(**beamSrc**.**EnergyModeDisplayName **==** "6X"**)**

**{**

dlg_delta **=** dlg_delta_6X**;**

**}**

**if** **(**beamSrc**.**EnergyModeDisplayName **==** "6X-FFF"**)**

**{**

dlg_delta **=** dlg_delta_6F**;**

MachineParameters**.**PrimaryFluenceModeId **=** "FFF"**;**

**}**

var beamTmp **=** planNew**.**AddVMATBeam**(**MachineParameters**,** mwSrc**,** collimatorAngle**,** ganSrc**.**First**(),**

ganSrc**.**Last**(),** gantryDirection**,** 0.0**,** isocenter**);** // automatically inserts corresponding MLC

// temporary beam to generate interpolation factors

var ganNew **=** beamTmp**.**ControlPoints**.**Select**(**cp **=>** cp**.**GantryAngle**).**ToArray**();**

planNew**.**RemoveBeam**(**beamTmp**);**

int Ncp_s **=** ganNew**.**Length**;**

double**[]** Gp_src **=** **new** double**[**Ncp_s**];** Gp_src**[**0**]** **=** 0**;**

double**[]** Gd_src **=** **new** double**[**Ncp_s**];** // difference between adjacent elements

double Gdiff **=** 0**;**

double pf **=** 1**;** // progress factor

**if** **(**gantryDirection**.**Equals**(**VMS**.**TPS**.**Common**.**Model**.**Types**.**GantryDirection**.**CounterClockwise**))**

**{**

pf **=** **-**1**;**

**}**

// generate interpolation for gantry angles

**for** **(**int cpIdx **=** 1**;** cpIdx **<** Ncp_s**;** cpIdx**++)** // iterate over src beam control points

**{**

// create gantry progress vector for src beam

Gdiff **=** pf ***** **(**ganSrc**[**cpIdx**]** **-** ganSrc**[**cpIdx **-** 1**]);**

**if** **(**Gdiff **<** 0**)** **{** Gdiff **+=** 360**;** **}**

Gd_src**[**cpIdx**]** **=** Gdiff**;**

Gp_src**[**cpIdx**]** **=** Gp_src**[**cpIdx **-** 1**]** **+** Gdiff**;**

**}**

int Ncp_n **=** ganNew**.**Length**;**

double**[]** Gp_new **=** **new** double**[**Ncp_n**];** Gp_new**[**0**]** **=** 0**;**

double Gp_ni **=** 0**;**

int idxL **=** 0**;**

int maxIdx **=** Ncp_s **-** 1**;**

double intStep **=** 0**;**

int**[]** intpIdx **=** **new** int**[**Ncp_n**];**

float**[]** intpStp **=** **new** float**[**Ncp_n**];**

int**[]** intp_md **=** **new** int**[**Ncp_n**];**

double**[]** mwNew **=** **new** double**[**Ncp_n**];**

**for** **(**int cpIdx **=** 0**;** cpIdx **<** Ncp_n**;** cpIdx**++)** // iterate over new beam's control points

**{**

**if** **(**cpIdx **>** 0**)**

**{**

Gdiff **=** pf ***** **(**ganNew**[**cpIdx**]** **-** ganNew**[**cpIdx **-** 1**]);**

**if** **(**Gdiff **<** 0**)** **{** Gdiff **+=** 360**;** **}**

Gp_ni **=** Gp_new**[**cpIdx **-** 1**]** **+** Gdiff**;**

**}**

**else**

**{**

Gdiff **=** pf ***** **(**ganNew**[**cpIdx**]** **-** ganSrc**[**cpIdx**]);**

**if** **(**Gdiff **<** 0**)** **{** Gdiff **+=** 360**;** **}**

Gp_ni **=** Gdiff**;**

**}**

Gp_new**[**cpIdx**]** **=** Gp_ni**;**

// compare Gp_ni with indices

**while** **((**idxL **<** maxIdx**)** **&&** **(**Gp_ni **>** Gp_src**[**idxL**]))**

**{**idxL**++;}**

**if** **(**idxL **>** 0**)**

**{**idxL**--;}**

intp_md**[**cpIdx**]** **=** 0**;**

intpIdx**[**cpIdx**]** **=** idxL**;**

intStep **=** **(**Gp_ni **-** Gp_src**[**idxL**])** **/** **(**Gp_src**[**idxL **+** 1**]** **-** Gp_src**[**idxL**]);**

intpStp**[**cpIdx**]** **=** **(**float**)**intStep**;**

mwNew**[**cpIdx**]** **=** mwSrc**[**idxL**]** **+** intStep ***** **(**mwSrc**[**idxL **+** 1**]** **-** mwSrc**[**idxL**]);**

**}**

IEnumerable**<**double**>** IE_mwNew **=** mwNew**;**

var beamNew **=** planNew**.**AddVMATBeam**(**MachineParameters**,** IE_mwNew**,** collimatorAngle**,** ganSrc**.**First**(),**

ganSrc**.**Last**(),** gantryDirection**,** 0.0**,** isocenter**);**

**if** **(**beamNew**.**Id**.**Length **<** 15**)**

**{**beamNew**.**Id **=** beamSrc**.**Id **+** " H"**;}**

var editableParams **=** beamNew**.**GetEditableParameters**();**

var CPnew **=** beamNew**.**ControlPoints**;**

var CPsrc **=** beamSrc**.**ControlPoints**;**

float intStepF**;**

double X1n**,** X2n**,** Y1n**,** Y2n**;**

float**[]** diffA **=** **new** float**[**60**];**

float**[]** diffB **=** **new** float**[**60**];**

float**[,]** MLC120L **=** **new** float**[**2**,** 60**];**

float**[,]** MLC120U **=** **new** float**[**2**,** 60**];**

float minA_gbl **=** 9999**;** float maxB_gbl **=** **-**9999**;**

**for** **(**int cpIdx **=** 0**;** cpIdx **<** Ncp_n**;** cpIdx**++)**

**{**

MLC120L **=** CPsrc**[**cpIdx**].**LeafPositions**;**

**if** **(**cpIdx **<** **(**Ncp_n **-** 1**))**

**{**

MLC120U **=** CPsrc**[**cpIdx **+** 1**].**LeafPositions**;**

**for** **(**int LfIdx **=** 0**;** LfIdx **<** 60**;** LfIdx**++)** // HDMLC index

**{**

**if** **(**diffA**[**LfIdx**]** **==** 0**)**

**{**

**if** **(**MLC120U**[**1**,** mapL**[**LfIdx**]]** **!=** MLC120L**[**1**,** mapL**[**LfIdx**]])**

**{**diffA**[**LfIdx**]** **=** 1**;}**

**}**

**if** **(**diffB**[**LfIdx**]==**0**)**

**{**

**if** **(**MLC120U**[**0**,** mapL**[**LfIdx**]]** **!=** MLC120L**[**0**,** mapL**[**LfIdx**]])**

**{**diffB**[**LfIdx**]** **=** 1**;}**

**}**

**}**

**}**

**for** **(**int LfIdx **=** 0**;** LfIdx **<** 60**;** LfIdx**++)** // HDMLC index

**{**

**if** **(**MLC120L**[**0**,** LfIdx**]** **>** maxB_gbl**)**

**{**maxB_gbl **=** MLC120L**[**0**,** LfIdx**];}**

**if** **(**MLC120L**[**1**,** LfIdx**]** **<** minA_gbl**)**

**{**minA_gbl **=** MLC120L**[**1**,** LfIdx**];}**

**}**

**}**

// edit control points of new beam

**for** **(**int cpIdx **=** 0**;** cpIdx **<** Ncp_n**;** cpIdx**++)**

**{**

float**[,]** HDMLC **=** **new** float**[**2**,** 60**];**

idxL **=** intpIdx**[**cpIdx**];**

var JPsrcL **=** CPsrc**[**idxL**].**JawPositions**;**

intStepF **=** intpStp**[**cpIdx**];**

MLC120L **=** CPsrc**[**idxL**].**LeafPositions**;**

MLC120U **=** CPsrc**[**idxL **+** 1**].**LeafPositions**;**

var JPsrcU **=** CPsrc**[**idxL **+** 1**].**JawPositions**;**

X1n **=** JPsrcL**.**X1 **+** intStepF ***** **(**JPsrcU**.**X1 **-** JPsrcL**.**X1**);**

X2n **=** JPsrcL**.**X2 **+** intStepF ***** **(**JPsrcU**.**X2 **-** JPsrcL**.**X2**);**

Y1n **=** JPsrcL**.**Y1 **+** intStepF ***** **(**JPsrcU**.**Y1 **-** JPsrcL**.**Y1**);**

Y2n **=** JPsrcL**.**Y2 **+** intStepF ***** **(**JPsrcU**.**Y2 **-** JPsrcL**.**Y2**);**

**if** **(**Y1n **>** 110 **||** Y2n **>** 110**)**

**{throw** **new** ApplicationException**(**"Y jaw > 11cm; ensure isocentre is balanced SUP-INF"**);}**

**for** **(**int LfIdx **=** 0**;** LfIdx **<** 60**;** LfIdx**++)**

**{**

// remap leaves (without DLG correction)

HDMLC**[**0**,** LfIdx**]** **=** MLC120L**[**0**,** mapL**[**LfIdx**]]** **+** intStepF ***** **(**MLC120U**[**0**,** mapL**[**LfIdx**]]** **-** MLC120L**[**0**,** mapL**[**LfIdx**]]);**

HDMLC**[**1**,** LfIdx**]** **=** MLC120L**[**1**,** mapL**[**LfIdx**]]** **+** intStepF ***** **(**MLC120U**[**1**,** mapL**[**LfIdx**]]** **-** MLC120L**[**1**,** mapL**[**LfIdx**]]);**

// screen for whether DLG correction should be applied

**if** **((**diffA**[**LfIdx**]** **>** 0**)** **&&** **(**diffB**[**LfIdx**]** **>** 0**))**

**{**

**if** **((**HDMLC**[**1**,** LfIdx**]** **-** HDMLC**[**0**,** LfIdx**])** **>** 0.6f**)**

**{**

// compare with globally most extended

**if** **((**maxB_gbl **-** HDMLC**[**0**,** LfIdx**]** **+** dlg_delta**)** **<** eps**)**

**{**

HDMLC**[**0**,** LfIdx**]** **=** HDMLC**[**0**,** LfIdx**]** **-** dlg_delta**;**

**}**

**if** **((**HDMLC**[**1**,** LfIdx**]** **+** dlg_delta **-** minA_gbl**)** **<** eps**)**

**{**

HDMLC**[**1**,** LfIdx**]** **=** HDMLC**[**1**,** LfIdx**]** **+** dlg_delta**;**

**}**

**}**

**}**

**}**

**if** **(**Y1n **<** **-**105**)**

**{**Y1n **=** **-**105**;}**

**if** **(**Y2n **>** 105**)**

**{**Y2n **=** 105**;}**

//editableParams.ControlPoints.ElementAt(cpIdx).JawPositions = CPsrc[idxL].JawPositions;

editableParams**.**ControlPoints**.**ElementAt**(**cpIdx**).**JawPositions **=** **new** VRect**<**double**>(**X1n**,** Y1n**,** X2n**,** Y2n**);**

editableParams**.**ControlPoints**.**ElementAt**(**cpIdx**).**LeafPositions **=** HDMLC**;**

**}**

editableParams**.**WeightFactor **=** beamWeight**;**

beamNew**.**ApplyParameters**(**editableParams**);**

planNew**.**RemoveBeam**(**planNew**.**Beams**.**Where**(**o **=>** o**.**Id **==** beamSrc**.**Id**).**SingleOrDefault**());**

**}**

**}**

MessageBox**.**Show**(**string**.**Format**(**"HDMLC plan {0} created in course {1}."**,** planNew**.**Id**,** course**.**Id**));**

**}**

**}**

**}**
